# Toward sustainable crops: integrating vegetative (non-seed) lipid storage, carbon-nitrogen dynamics, and redox regulation

**DOI:** 10.3389/fpls.2025.1589127

**Published:** 2025-06-03

**Authors:** Somrutai Winichayakul, Nick Roberts

**Affiliations:** Resilient Agriculture, AgResearch Ltd., Palmerston North, New Zealand

**Keywords:** C/N partitioning, non-seed lipid storage, redox regulation, sustainable agriculture, bioenergy, abiotic stress

## Abstract

The global challenges of climate change and rising energy demands necessitate innovative agricultural solutions. One promising strategy is the transformation of photosynthetic tissues into lipid-rich organs, providing energy-dense biomass for biofuel production while enhancing carbon sequestration. However, these metabolic shifts require substantial NADPH and ATP, reshaping cellular processes such as the Calvin-Benson cycle, glycolysis, and oxidative pentose phosphate pathways. This review explores the intricate metabolic and regulatory networks underpinning lipid accumulation, with a focus on carbon/nitrogen partitioning, redox regulation, and their implications for plant stress tolerance and productivity. Furthermore, we highlight recent progress in field applications, multi-omics integration, and emerging strategies to optimize lipid accumulation in crops while mitigating trade-offs in biomass yield and agronomic performance. Understanding these complex interactions will be essential for developing sustainable, high-lipid crops that support bioenergy production and climate-resilient agriculture.

## Highlights

- The review explores the intricate metabolic and regulatory networks underlying lipid accumulation in non-seed tissues, emphasizing carbon/nitrogen partitioning, redox regulate on, and their impact on plant stress tolerance and productivity.
- Key future research directions are outlined, including integrative multi-omics approaches, environmental stress adaptation, carbon-nitrogen interactions, and extensive field trials.
- The potential of alternative crop species and systems is discussed, highlighting opportunities to broaden the applications of these technologies for sustainable agriculture and bioenergy production.

## 1 Introduction

The growing global demand for food, forage, and biofuels calls for innovative strategies to enhance crop productivity. One promising approach is increasing lipid biosynthesis in non-seed tissues, offering a dual benefit: boosting the energy density of plant biomass and reducing dependency on carbohydrate-dominated metabolism (Heaton et al., 2008; Vanhercke et al., 2019). Triacylglycerol (TAG) is an energy-dense carbon (C) sink, storing over twice as much energy per gram as carbohydrates, making it ideal for biofuel and feedstock production (Durrett et al., 2008; Xu and Shanklin, 2016).

Bioenergy feedstocks offer a solution to climate adaptation and sustainable energy challenges. Oils, being hydrophobic, help maintain cellular integrity under drought or heat stress, thereby enhancing the plant’s overall resilience (Lu et al., 2020; Perlikowski et al., 2022). Additionally, increasing vegetative oil storage can boost the plant’s C sink capacity, contributing to reduced net greenhouse gas emissions when these crops are cultivated at scale (Paul and Eastmond, 2020; Beechey-Gradwell et al., 2020). Moreover, leaf oil-rich biomass can serve as a dual-purpose feedstock, supporting biofuel production while simultaneously providing valuable co-products, such as high-energy livestock feed (Winichayakul et al., 2020; Beechey-Gradwell et al., 2022).

In this review, when referring to “high-lipid plants”, we specifically mean vegetative oil storage plants—those that accumulate significant amounts of lipids in their non-seed tissues, such as leaves, stems, or roots. Unlike traditional oilseed crops, which store lipids primarily in seeds, these high-lipid plants are engineered to store oils in their vegetative tissues, making them a novel and promising resource for various applications in bioenergy, biofuels, and livestock feed. For examples, high-lipid perennial ryegrass (*Lolium perenne*) has been engineered to enhance forage energy efficiency, reducing reliance on expensive lipid supplements (e.g., oilseeds, vegetable oils) while improving the nutritional quality of animal-derived products (Bayat et al., 2018; Beechey-Gradwell et al., 2022). Beyond forage applications, other high-biomass species—such as tobacco (*Nicotiana tabacum*) and C4 sorghum (*Sorghum bicolor*)—have been targeted for sustainable bioenergy production due to their resilience under abiotic stresses (e.g., drought, heat) and capacity for lipid engineering in grains, stems, and leaves (Mullet et al., 2014; Vanhercke et al., 2018 & 2019). These modifications aim to optimize lipid yields for industrial applications, including biodiesel, biolubricants, and aviation fuels. **Table 1** summarizes the lipid content improvements, key genetic transformation events, and target applications for these engineered crops, highlighting their diverse roles in agriculture and bioeconomy.

**Table 1:** Engineered high-lipid crops: Targets, modifications, and applications.

Species	Tissue modified	Baseline lipid content	Engineered lipid content	Key genetic modifications	Primary applications	Citations
Arabidopsis	Root, stem, leaf	TFA; < 2% DW (root), 3-4% DW (leaf)	TAG; up to 5-8% DW (root, stem, leaf), TFA; up to 11% DW (leaf)	*DGAT1* + *Cys-OLEOSIN*; *adg1* + *DGAT1* + *OLEOSIN* + *suc2* + *WRI1*; *adg1* + *pxa1*; *adg1* + *sdp1*	Proof-of-concept for vegetative lipid storage	Kelly et al., 2013; Winichayakul et al., 2013; Zhai et al., 2017; Vanhercke et al., 2019.
Duckweeds	Frond	TFA; up to 7% under nitrogen limitation	TFA; 20-35% DW, TAG; 8.7% DW	*WRI1 + OLEOSIN + DGAT2 +* 100 µM estradiol	Biofuels, Aquafeed, Bioremediation	Liang et al., 2022; Yang, 2022.
Maize (corn)	Stover	TFA; 1% DW, TAG; 0.1% DW (leaf)	TFA; 2% DW, TAG; 0.15% DW	*DGAT1 + OLEOSIN + WRI1*	Biofuels, livestock feed, bio-based plastic, paper pulp	Alameldin et al., 2017
Potato	Tuber	TAG; < 0.1% DW	TAG; 3.3% DW	*DGAT1 +OLEOSIN + WRI1*	Nutritional value for human food and industrial uses	Liu et al., 2016.
Perennial ryegrass	Stem, leaf	TFA; 2-3% DW (stem), 3-4.5% (leaf)	TFA; up to 4% DW (stem), up to 8% DW (leaf)	*DGAT1* + *Cys-OLEOSIN*	Forage energy enhancement, livestock nutrition	Beechey-Gradwell et al., 2020 & 2022, Winichayakul et al., 2020
Sorghum	Seed, stem, leaf	3-4% DW (seed), < 2% DW (stem)	8-12% DW (seed), 5-6% DW (stem) TAG; up to 8.4% DW (leaf)	*LEC2* + *WRI1*; *fad2*; *DGAT2* + *OLEOSIN* + *WRI1*	Biofuels (biodiesel, SAF), dual-use food/feed	Mullet et al., 2014; Vanhercke et al., 2018; Park et al., 2025.
Sugarcane	Stem, leaf	TFA; 3-4 %DW (leaf)	TAG; 0.9% DW (stem), up to 4.4% DW (leaf)	*adg1 + DGAT1-2 + OLEOSIN + pxa1 + WRI1*	High biomass crop, biodiesel	Zale et al., 2016; Parajuli et al., 2020; Kannan et al., 2022.
Tobacco	Stem, leaf, whole plant	TFA; up to 5% DW (leaf)	TAG; 7.4% DW (stem) TFA; up to 17.7% DW (leaf), TAG; up to 15.8% DW (leaf)	*DGAT1* + *WRI1 + sdp1; DGAT1 + OLEOSIN + WRI1*	Biodiesel, biolubricants, industrial oils	Vanhercke et al., 2014; Zale et al., 2016; Vanhercke et al., 2017; Vanhercke et al., 2019.

*adg1*, ADP-glucose pyrophosphorylase 1; *DGAT1 & 2*, acyl-CoA diacylglycerol acyltransferase1 & 2; DW, dried weight; *fad2*, FA desaturase 2; *LEC2*, leafy cotyledon 2; *pxa1*, peroxisome membrane-associated D-type ABC transporter protein; *sdp1*, sugar-dependent 1 lipase; *suc2*, sucrose transporter involved in phloem loading; TAG, triacylglycerol; TFA, total fatty acids; *WRI1*, wrinkled 1.

Recent biotechnological advances have enabled the introduction and optimization of TAG biosynthesis pathways in these vegetative tissues, transforming them into efficient lipid-accumulating organs. This process requires the coordinated regulation of multiple biological processes, including chloroplastic *de novo* fatty acid (FA) biosynthesis, TAG assembly in the endoplasmic reticulum (ER), lipid droplet (LD) formation, and cytoplasmic LD storage (reviewed in Vanhercke et al., 2019). Additionally, significant progress has been made in redirecting C flux away from starch and sucrose biosynthesis and toward oil biosynthesis pathways (**Table 1**), further enhancing lipid yields (Sanjaya et al., 2011; Zhai et al., 2017).

However, redirecting C flux to lipid biosynthesis demands significant NADPH and ATP, affecting pathways like the Calvin-Benson cycle, glycolysis, and the oxidative pentose phosphate pathway (OxPPP) (Li-Beisson et al., 2013). Additionally, C and nitrogen (N) partitioning are tightly interconnected, with N assimilation playing a pivotal role in photosynthetic efficiency and plant growth (Bloom et al., 1985; Nunes-Nesi et al., 2010). Redox balance and C/N allocation regulate carbohydrate and lipid production, influencing how plants respond to their environment (Chaput et al., 2020).

This review examines the intricate metabolic and regulatory networks governing vegetative lipid biosynthesis, focusing on the interplay of C/N partitioning, energy metabolism, and redox regulation. We discuss how these dynamics shape plant responses to environmental conditions and highlight the potential of field applications to address global food and energy demands. Additionally, advancing integrative multi-omics approaches, understanding the role of lipid accumulation in stress adaptation, and conducting extensive field trials will be pivotal for scaling up these technologies and validating their benefits in real-world agricultural systems.

## 2 Coordination of carbon partitioning in high-lipid plants

Efficient C partitioning is a cornerstone of plant productivity, with far-reaching implications for agriculture, particularly in enhancing crop yield, stress tolerance, and resource use efficiency. In high-lipid plants, C partitioning is tightly regulated to balance the demands of growth, storage, and stress responses. This section explores the regulatory networks governing C flow in plants, emphasizing triose phosphate/phosphate translocator (TPT), glycolysis, and lipid biosynthesis, and their roles in optimizing vegetative lipid accumulation.

### 2.1 Triose phosphate transport and carbon partitioning

**Figure 1:**
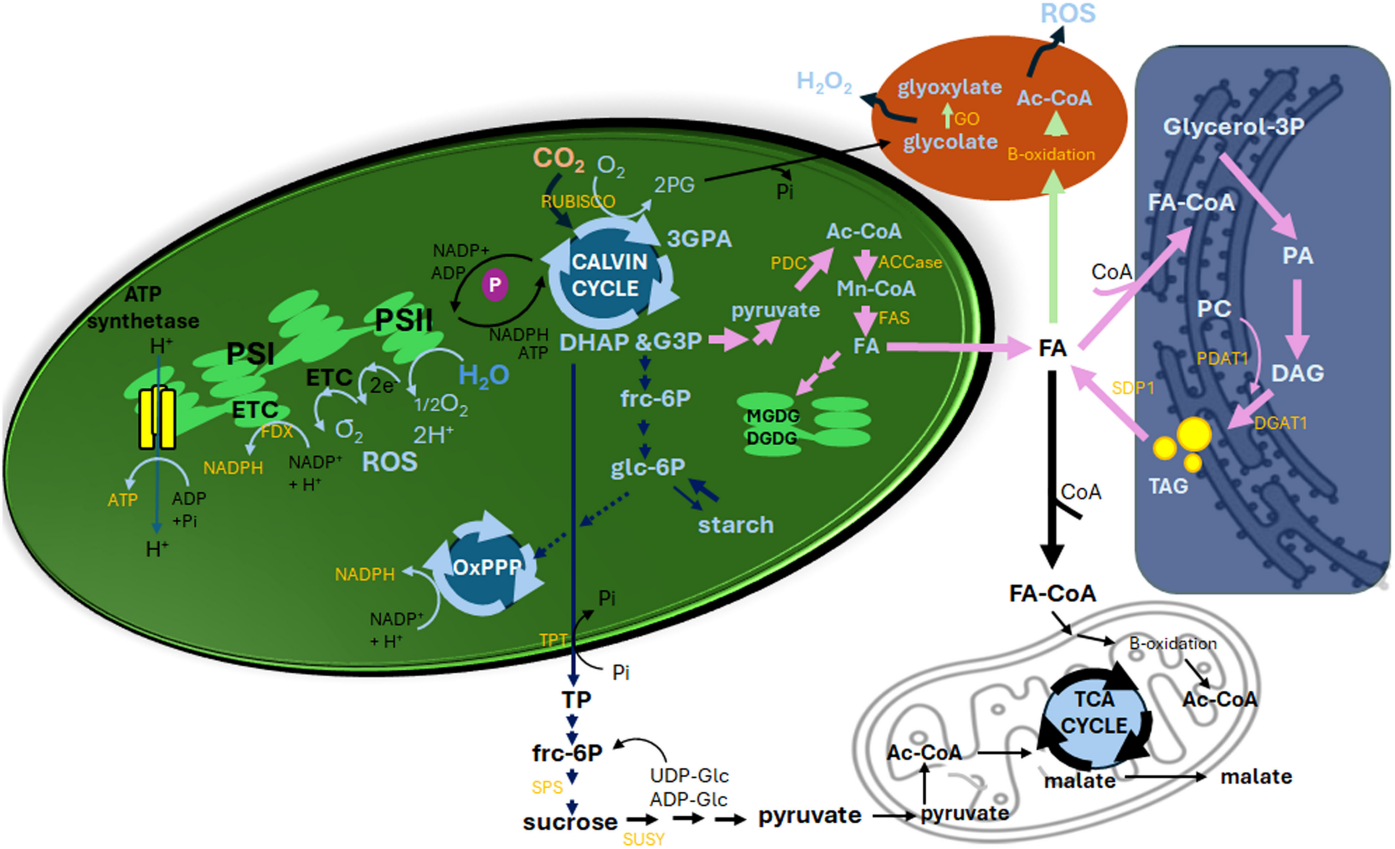
Interplay between photosynthesis, carbon metabolism, lipid biosynthesis, and redox homeostasis in high-lipid plant cells. Upon illumination, photosystem II (PSII) captures light energy, initiating the photolysis of water (H_2_O) and releasing oxygen (O_2_). Reactive oxygen species (ROS) are generated by the chloroplast electron transport chain (ETC), producing _1/2_O_2_ at PSII and O_2_^-^ **^·^** at PSI. PSI also facilitates ATP synthesis and generates NADPH via ferredoxin (FDX)-NADP reductase. These products fuel the Calvin cycle, where CO_2_ is fixed by ribulose-1,6-bisphosphate carboxylase/oxygenase (RUBISCO) to form 3-phosphoglyceric acid (3-PGA). Using ATP and NADPH, 3-PGA is reduced to glyceraldehyde-3-phosphate (G3P) and dihydroxyacetone phosphate (DHAP), collectively termed triose phosphates (TPs). During photorespiration, when O_2_ levels are high and CO_2_ levels are low, RUBISCO produces 2-phosphoglycolate (2PG) in coupling with the release of inorganic phosphate (Pi). This is transported to peroxisomes, where its metabolism by glycolate oxidase (GO) generates H_2_O_2_. When carbon metabolism favours sugar and starch synthesis, two G3P molecules form six-carbon fructose-6-phosphate (frc-6P) and glucose-6-phosphate (glc-6P) with total 18 ATP and 12 NADPH consumption for starch synthesis in the chloroplasts or TPs may be transported to the cytosol *via* the TP/phosphate translocator (TPT) and converted to sucrose by sucrose phosphate synthase (SPS) with an exchange to the cytosol Pi. Starch synthesized during the day is degraded at night into sucrose for energy needs. In high-lipid plants, carbon flux may shift toward lipid biosynthesis. G3P undergoes glycolysis, producing pyruvate, which is converted to acetyl-CoA (Ac-CoA) by the pyruvate dehydrogenase complex (PDC). Ac-CoA is carboxylated to malonyl-CoA by acetyl-CoA carboxylase (ACCase), the first step in fatty acid (FA) synthesis. This process requires NADPH, and the resulting FAs follow multiple pathways: FAs undergo multiple pathways: (1) Membrane Lipid Synthesis: FAs are incorporated into monogalactosyldiacylglycerol (MGDG) and digalactosyldiacylglycerol (DGDG). (2) β-Oxidation in Mitochondria: FAs are activated to FA-CoA and transported via the carnitine shuttle for oxidation, yielding Ac-CoA, NADH, and FADH_2_. Ac-CoA enters the TCA cycle, while NADH and FADH_2_ fuel the mitochondrial ETC. (3) Glycerolipid Assembly: FA-CoA derivatives participate in forming phosphatidic acid (PA), phosphatidylcholine (PC), diacylglycerol (DAG), and triacylglycerol (TAG), involving Ac-CoA: diacylglycerol acyltransferase 1 (DGAT1) and phosphatidylcholine: diacylglycerol acyltransferase 1 (PDAT1) activities. TAG turnover is dynamic, with sugar-dependent lipase (SDP1) mediating its degradation. (4) β-Oxidation in Peroxisomes: This process regenerates Ac-CoA and releases ROS, which, if exceeding catalase capacity, may contribute to oxidative stress. Sucrose breakdown by sucrose synthase (SUSY) produces UDP-glucose and fructose, fuelling glycolysis to generate pyruvate, ATP, and NADH. A portion of glucose and fructose enters the oxidative pentose phosphate pathway (OxPPP) to supply NADPH for FA biosynthesis and redox balance. Pyruvate also enters the TCA cycle, generating intermediates like malate and citrate. Malate may be exported between mitochondria and cytosol through the malate-aspartate shuttle, balancing redox states *via* NADH transfer. Arrows may indicate reactions involving more than one step. Thick arrows indicate pathways favoured in high-lipid plants, highlighting metabolic flexibility and integration.

Dihydroxyacetone phosphate and glyceraldehyde 3-phosphate, key triose phosphate (TP) metabolites, play a crucial role in C partitioning between starch, sucrose, and lipids (**Figure 1**). Under high photosynthetic activity, increased TP levels coincide with decreased inorganic phosphate (Pi) availability, as Pi is consumed in ATP synthesis. TPT, an antiporter located at the inner chloroplast membrane envelope, exchanges stromal TPs with cytosolic Pi, facilitating the distribution of photosynthetic C from chloroplasts to the cytosol (Fliege et al., 1978). When sink tissues require fewer exported carbohydrates than the photosynthesis rate generates, or when Pi and sucrose synthesis rates are limited, TPs are temporarily stored as starch (Zeeman et al., 2002). Optimizing TPT to enhance sucrose export while maintaining adequate starch reserves could potentially boost biomass and improve stress resilience (Schneider et al., 2002).

Interestingly, impaired TPT function does not impair growth under normal conditions. However, overexpression of TPT and cytosolic fructose-1,6-bisphosphatase activates sucrose-phosphate synthase (SPS), accelerating sucrose synthesis and promoting growth (Huber and Huber, 1992; Cho et al., 2012). In rice mutants with reduced SPS activity (84% decrease), starch content increased, but growth remained unaffected, suggesting that higher SPS activity promotes more efficient TP export, limiting C availability for starch synthesis (Hashida et al., 2016). RNA interference-mediated downregulation of ADP-glucose pyrophosphorylase, a key enzyme in starch biosynthesis, resulted in a dramatic 16-fold increase in TAG and other lipid accumulation in both wild-type and high-oil potato lines (Xu et al., 2019). This metabolic shift was accompanied by substantial alterations in sugar profiles and starch content across both tuber and leaf tissues, along with significant changes in tuber starch properties. The study demonstrates how redirecting C flux from starch synthesis toward lipid biosynthesis can dramatically enhance oil accumulation in vegetative tissues. These findings underscore the critical importance of balancing starch and sucrose synthesis to maintain C availability for lipid production, as these competing pathways both depend on photoassimilates generated by the Calvin cycle (Sanjaya et al., 2011).

### 2.2 Glycolysis and lipid biosynthesis

Glycolysis is a fundamental metabolic pathway linking C partitioning, providing substrates for FA biosynthesis *via* acetyl-CoA carboxylase (ACCase) and the FA synthase (FAS) complex in the chloroplasts (**Figure 1**, pink arrows). Cytosolic pyruvate, derived from glycolysis, feeds into FA synthesis within plastids, where plastidic pyruvate is converted to acetyl-CoA (Ac-CoA) by the pyruvate dehydrogenase complex (PDC). This tightly regulated process ensures sufficient Ac-CoA for FA biosynthesis. Studies in tobacco using ¹³CO_2_ labelling and metabolic flux analysis reveal that starch and sucrose cycling support lipid biosynthesis demands (Chu et al., 2022). In high-lipid ryegrass lines, lower shoot sugar levels were associated with reduced fructan biosynthesis and upregulated PDC transcripts (Winichayakul et al., 2022).

Feedback mechanisms further highlight the interconnectedness of glycolysis and lipid biosynthesis. In high-lipid ryegrass, upregulated hexokinase expression suggests enhanced glycolysis, providing substrates for pyruvate and Ac-CoA production (Winichayakul et al., 2022). Elevated hexose phosphate levels may also fuel the OxPPP, the primary source of lipogenic NADPH, as shown by ¹³C metabolic flux analysis in oleaginous yeast cells (Zhang et al., 2016; Kamineni and Shaw, 2020) and biofuel-relevant industrial fungi (Masi et al., 2021). The coordination between glycolysis, the OxPPP, and lipid biosynthesis represents a critical metabolic nexus in high-lipid plants, fundamentally governed by ATP and NADPH requirements. Glycolytic flux begins with glucose-6-phosphate (Glc-6P) from sucrose breakdown, which enters both glycolysis and OxPPP, yielding pyruvate (a precursor for plastidic Ac-CoA), ATP (energy for FA elongation), and NADH (fed into the mitochondrial electron transport chain (ETC)), with key regulatory points including hexokinase activation in high-lipid ryegrass (Winichayakul et al., 2022) and plastidic PDC upregulation (Chu et al., 2022). Meanwhile, the OxPPP plays a dual role as the primary NADPH generator (producing four molecules per Glc-6P) and maintains redox balance during lipid synthesis, supplying NADPH (**Figure 1**, Li-Beisson et al., 2013). This intricate interplay ensures efficient C allocation and energy provision for lipid biosynthesis.

### 2.3 Malate and pyruvate reactions in high-lipid plants

**Figure 2:**
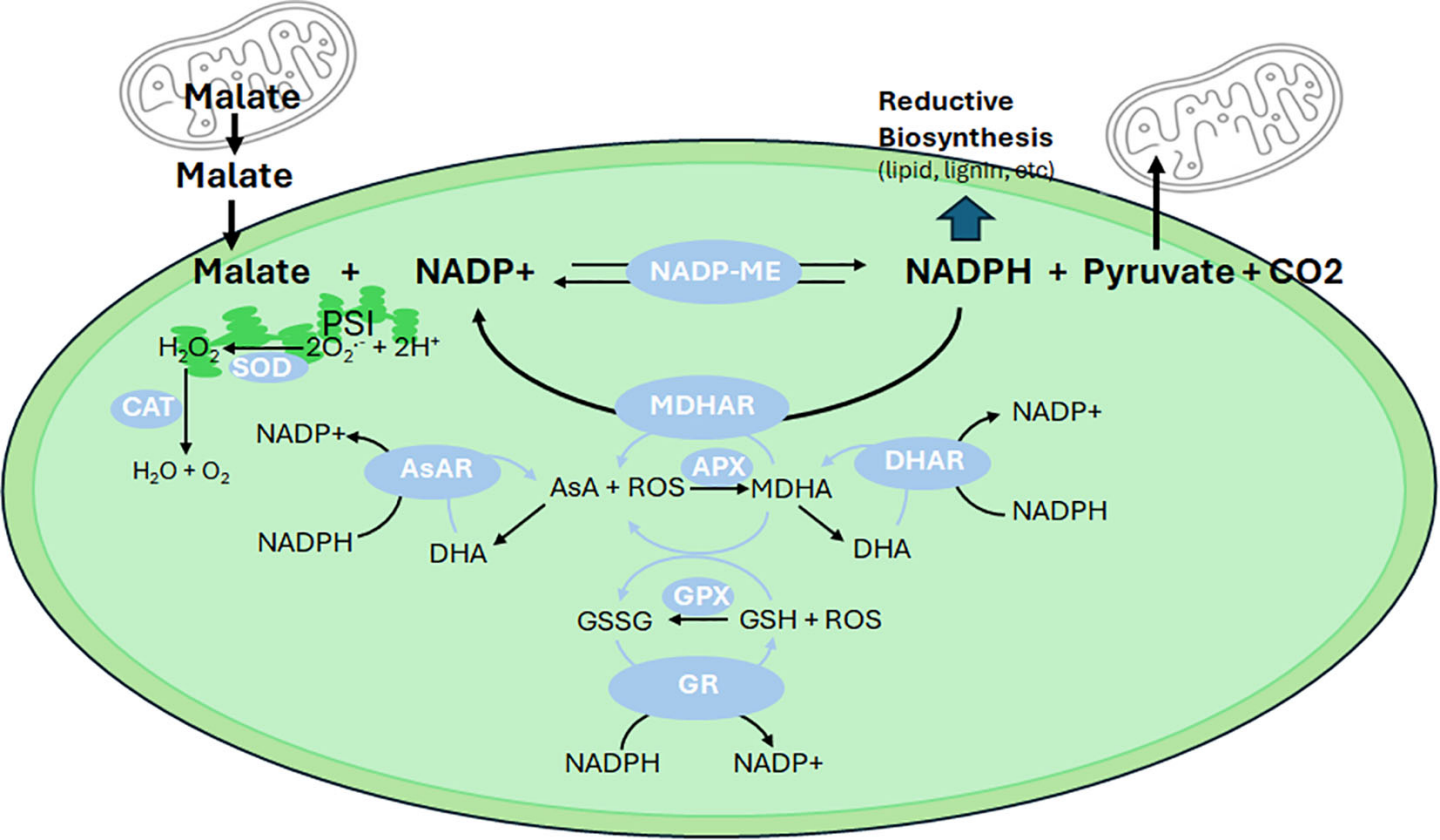
Role of NADP-malic enzyme and the SOD-AsA-GSH cycle in redox balance. Malate exported from the mitochondria to the cytosol is subsequently transported into chloroplasts, where it undergoes decarboxylation by NADP-malic enzyme (NADP-ME). This reaction generates NADPH, pyruvate, and CO_2_. NADPH plays a vital role in biosynthetic processes, such as the production of fatty acids, flavonoids, and lignin, while pyruvate can be transported back to the mitochondria for further metabolic functions as shown in **Figure 1**. NADPH also directly supports the ascorbate (AsA)-glutathione (GSH) cycle, which is crucial for detoxifying reactive oxygen species (ROS) and maintaining redox homeostasis. In this cycle, monodehydroascorbate (MDHA) is reduced to AsA by MDHA reductase (MDHAR) using NADPH as the reducing power. AsA, through the action of ascorbate peroxidase (APX), scavenges ROS and is subsequently oxidized to form MDHA and dehydroascorbate (DHA). DHA is then rapidly reduced back to MDHA by DHA reductase (DHAR) or to AsA by ascorbate reductase (AsAR), both utilizing NADPH as an electron donor. Furthermore, glutathione (GSH) plays a role in ROS detoxification by glutathione peroxidase (GPX), which converts GSH to its oxidized form, GSSG. Glutathione reductase (GR) then regenerates GSH from GSSG using NADPH. GSH can also directly reduce MDHA to AsA, highlighting its integral role in the AsA-GSH cycle. Photochemically generated ROS, such as superoxide (O_2_^-^ **^·^**), is converted to hydrogen peroxide (H_2_O_2_) by superoxide dismutase (SOD). The resulting H_2_O_2_ is either reduced to water (H_2_O) via the AsA-GSH cycle or decomposed by catalase (CAT).

Cytosolic pyruvate supports mitochondrial tricarboxylic acid cycle activity, producing C4 compounds like malate (**Figure 1**). Malate facilitates the transfer of reducing equivalents between the cytosol and mitochondria *via* the malate-aspartate shuttle and can be exported to the chloroplast through the “malate valve” (Selinski and Scheibe, 2019). NADP-malic enzyme (NADP-ME) activity, through malate decarboxylation, maintains metabolic flexibility and redox balance by producing pyruvate and NADPH (**Figure 2**). Recent studies demonstrate that NADP-ME activity not only supplies NADPH for lipid biosynthesis but also mitigates oxidative stress by regenerating NADP^+^, essential for sustaining photosynthetic electron flow (Chu et al., 2022; Winichayakul et al., 2025). This is particularly relevant in high-lipid plant leaves, where manipulation of NADP-ME activity in tobacco and Arabidopsis increased leaf lipid content by up to 30%, demonstrating its potential as a target for metabolic engineering (Wheeler et al., 2005; Chu et al., 2022). In these studies, ¹³CO_2_ metabolic flux modelling and acyl-acyl carrier protein analysis confirmed that starch production, sucrose cycling, and NADP-ME contribute significantly to lipid synthesis. Enzyme activity assays further support this role. Additionally, NADP-ME activity helps maintain redox balance by regenerating NADP^+^, which is essential for sustaining photosynthetic electron flow and preventing ROS overproduction (**Figure 2**). Additionally, fluxes to Ac-CoA and FA production increased independently of lipid pool size data and without the need to impose constraints on lipid fluxes. These findings highlight the plant’s inherent capacity to establish a uniquely developmentally regulated carbon sink. Thus, manipulating NADP-ME activity could serve as a promising target for future efforts aimed at engineering high-lipid crops.

### 2.4 Regulatory role of WRINKLED1 and related factors

Transcription factors such as WRINKLED1 (WRI1) directly stimulate the expression of key enzymes in both the glycolytic and lipid biosynthesis pathways. WRI1 upregulates glycolytic enzymes to supply substrates for lipid production, with overexpression enhancing TAG accumulation and FA turnover (Sanjaya et al., 2011; Kuczynski et al., 2022). Recent discovered targets of WRI1 include enzymes in glycolysis and the pentose phosphate pathway, such as plastidic isoforms of fructokinase 3 and phosphoglucose isomerase 1 (Kuczynski et al., 2022). However, in combined with WRI1 expression, blocking starch synthesis or FA turnover negatively impacts FA accumulation (Fan et al., 2017). KIN10 is a catalytic subunit of the sucrose non-fermented 1 related-kinase 1 (SnRK1) complex, a central regulator of energy homeostasis in plants. Notably, WRI1 stability is modulated by trehalose-6-phosphate (T6P), which inhibited KIN10-mediated phosphorylation of WRI1, enhancing FA biosynthesis (Zhai et al., 2018). Synergistic interactions between WRI1 and TAG-synthesizing enzymes like Ac-CoA: diacylglycerol acyltransferase 1 (DGAT1) and phosphatidylcholine: diacylglycerol acyltransferase 1 (PDAT1) (**Figure 1**) have been leveraged to engineer oil-rich vegetative tissues and seeds (reviewed in Vanhercke et al., 2019). WRI1 synergizes with DGAT1 and PDAT1 by co-upregulating glycolytic genes (e.g., plastidic fructokinase) while DGAT1 channels fatty acids into TAG, avoiding cytotoxic free FA accumulation (Vanhercke et al., 2019). For example, co-expression of WRI1 and DGAT1 in tobacco increased leaf TAG by 15-fold compared to WRI1 alone (Zhou et al., 2020).

### 2.5 Impacts of carbon sink manipulation

Diverting C allocation from sugars to lipids impacts various metabolic pathways and subsequent growth, with outcomes depending on the species and lipid storage extent. In some plants, enhanced lipid storage improves water-use efficiency (WUE), photosynthetic N-use efficiency (NUE), and stress tolerance without growth penalties, although these improvements may not lead to more robust plants (Vanhercke et al., 2014; Beechey-Gradwell et al., 2018 & 2020). These benefits may arise from lipid C sinks mitigating photosynthetic feedback inhibition (Paul and Eastmond, 2020), with T6P and SnRK1 signaling pathways playing a crucial role in balancing sink limitation (Nunes et al., 2013). Elevated T6P levels signal sufficient sucrose availability, inhibiting SnRK1 activity and reducing photosynthetic C fixation to prevent overload (Tsai and Gazzarrini, 2014). In contrast, low energy levels activate SnRK1 to conserve resources, suppressing anabolic processes like photosynthesis (Baena-González and Lunn, 2020). Altered T6P/SnRK1 signaling complexes have been observed in high-lipid ryegrass, supporting this hypothesis (Winichayakul et al., 2022). Like high-lipid ryegrass, other high-lipid plants (Alameldin et al., 2017; Vanhercke et al., 2019; Kannan et al., 2022; Luo et al., 2022; Cao et al., 2023; Morales et al., 2024) exemplify the potential of manipulating C sinks to enhance agricultural energy density. However, excessive lipid biosynthesis may divert resources from essential processes such as membrane lipid and cell wall synthesis or protein production, potentially imposing growth penalties (Kelly et al., 2013; Beechey-Gradwell et al., 2020; Mitchell et al., 2020).

Independent of N availability, increased lipid content in non-photosynthetic tissues influences plant growth through multiple mechanisms. Lipids serve as energy-dense C reserves, providing ATP and C skeletons via β-oxidation during germination or stress recovery, supporting metabolic demands (Yang and Benning, 2018; Yang et al., 2022). In storage organs (e.g., seeds), high lipid content is adaptive and may not hinder growth if C supply is sufficient (Baud and Lepiniec, 2010). Lipids also play critical roles in membrane integrity and stress resilience; increased unsaturated FAs, for example, enhance membrane fluidity, sustaining cellular processes under abiotic stress (Dutta et al., 2025). Additionally, lipid-derived signaling molecules (e.g., jasmonates) modulate growth-defense trade-offs, potentially prioritizing stress responses over rapid growth (Wang et al., 2021). The overall impact on growth depends on C partitioning—non-photosynthetic tissues with high lipid content may act as strong C sinks, either supporting later growth phases (e.g., perennials) or limiting immediate structural growth if lipid synthesis outweighs carbohydrate allocation (Troncoso-Ponce et al., 2011; Schwender et al., 2015). Additionally, tobacco with >15% leaf TAG shows stunted growth due to resource competition with cell wall biosynthesis (Zhou et al., 2020). However, ryegrass engineered for moderate lipid accumulation (up to 6.5% DW) maintains biomass yield, suggesting species-specific adaptations further shape this relationship (Beechey-Gradwell et al., 2022; Theodoulou and Eastmond, 2012). Thus, the balance between lipid storage and growth hinges on C allocation strategies, tissue type, and environmental conditions, with trade-offs often observed in vegetative tissues but benefits in storage or stress-adapted organs (Cai and Shanklin, 2022).

To mitigate these trade-offs in vegetative tissues, strategies that integrate transcription factors and metabolic feedback and signaling controls are essential. For examples, engineering plants to enhance the expression of metabolite and regulatory proteins such as T6P, SnRK1, and TPT could optimize C partitioning, particularly when photosynthetic C supply is limited (Nunes et al., 2013; Tsai and Gazzarrini, 2014; Baena-González and Lunn, 2020). In addition, comparative studies using lipidomics, metabolic flux analyses, and multi-omics integration can reveal metabolic flexibility and subsequent regulatory differences and evaluate plant performance under different stresses across a range of high-lipid species (Peng et al., 2023; Liu et al., 2024). These approaches, coupled with field trial data and computational models, could offer insights into predicting plant productivity across diverse climatic conditions, informing field applications.

## 3 Nitrogen partitioning in high-lipid crops: a key aspect of sustainable crop improvement

N partitioning in high-lipid crops is critical for achieving high yields and sustainability goals. Optimizing photosynthetic NUE and understanding the interplay between N metabolism and lipid biosynthesis can reduce reliance on synthetic fertilizers, mitigate environmental impacts, and enhance economic viability. This section explores the role of N partitioning in photosynthetic efficiency, lipid biosynthesis, and stress responses, with a focus on how N availability and form influence plant performance.

### 3.1 Nitrogen demand in high-lipid crops

Leaf N levels are essential for maintaining photosynthetic efficiency, providing C precursors and energy, particularly during vegetative growth and under stress (Mengesha, 2021). Redistributing N toward vegetative lipid storage tissues may alter sink strength, affecting resource allocation between source and sink organs. For example, in high-lipid ryegrass, increased C allocation to vegetative oil enhances N uptake for growth and energy requirements, particularly under non-limiting N conditions (Beechey-Gradwell et al., 2018). This suggests that high-lipid crops may achieve higher photosynthetic NUE by optimizing N allocation between photosynthesis and lipid biosynthesis.

Assessing the dynamic C/N ratio and Rubisco’s maximum carboxylation capacity can help evaluate plant responses to N limitation (Cooney et al., 2021). Under N-deficient conditions, lipid accumulation often increases at the expense of growth, as seen in microalgae (Mata et al., 2010; Breuer et al., 2012). This trade-off highlights the need to balance N availability with lipid production to avoid growth penalties. For instance, in Arabidopsis, phospholipase Dϵ promotes growth and N signaling under severe N deprivation by increasing phosphatidic acid (PA) content, linking lipid metabolism to N stress responses (Hong et al., 2009).

### 3.2 Nitrogen partitioning in lipid storage vegetative tissues

Approximately 75% of leaf N is allocated to photosynthesis machinery, including Rubisco carboxylation (N_Rubisco_), bioenergetics (electron transport and ATP synthesis, N_E_), and light-harvesting pigment-protein complexes (N_P_) (Hikosaka and Terashima, 1995). N is also distributed in leaves in other forms (N_o_), such as soluble components (NO_3_^-^, NH_4_^+^, amino acids) and insoluble components (e.g., cell walls, membranes, and other structures) (Feng et al., 2009; Wei et al., 2022). Small changes in photosynthetic N allocation can significantly affect carboxylation efficiency and photosynthetic NUE (Feng et al., 2009; Onoda et al., 2017). For example, in high-lipid ryegrass, increased N allocation to N_E_ at the expense of N_O_ was observed under NO_3_^-^ treatment, without compromising N_Rubisco_ or N_P_ (Cooney et al., 2021). This optimal N allocation correlated with higher photosynthetic NUE, suggesting that high-lipid plants can adaptively balance nutrient allocation to achieve functional efficiency.

In high-lipid ryegrass, elevated leaf NO_3_^-^ under NO_3_^-^ supply suggests limited NO_3_^-^ ammonification or extensive nitrification of NH_4_^+^ in shoots, potentially mitigating oxidative stress from FA biosynthesis. Elevated shoot NO_3_^-^ has also been noted in other species as a local and systemic signal, regulating genome-wide gene expression and phytohormone signaling pathways through Ca²^+^ mediation (Alvarez et al., 2012).

### 3.3 Nitrogen form: the balance between growth and lipid biosynthesis

The form of N availability (NH_4_^+^ vs. NO_3_^-^) significantly influences growth, lipid accumulation, and N partitioning. Elevated atmospheric CO_2_ levels can inhibit NO_3_^-^ assimilation in C3 plants and algae, potentially reducing NO_3_^-^ use efficiency in future climates (Bloom, 2015 & 2020). However, this relationship is complex and remains a subject of debate. Andrews et al. (2019) discuss the variability of this response, noting that inhibition of N assimilation under elevated CO_2_ is not universal and depends on species-specific traits and N sources. While NH_4_^+^, is theoretically preferred due to its lower energy cost for assimilation, it can be toxic at high concentrations, necessitating strategies to improve NH_4_^+^ use efficiency (Di, 2023; Xiao et al., 2023).

In high-lipid ryegrass, shoot dry weight and FA content increased across N supply ranges, with no difference in response to N form at the T_0_ stage (Beechey-Gradwell et al., 2018). However, at the T_2_ stage, plants exhibited higher shoot growth rates under NH_4_^+^ than NO_3_^-^ at 20 mM concentrations, possibly due to genotypic background or non-limiting N conditions. In oleaginous fungi, NH_4_^+^ boosts lipid production due to its lower energy cost, redirecting resources toward FA synthesis (Wang et al., 2024b). Conversely, NO_3_^-^ prolongs N uptake by stimulating cytokinin production, delaying leaf senescence and maintaining root N uptake activity (Heuermann et al., 2021).

Crop-specific responses to N forms vary. For examples, Camelina prefers NO_3_^-^ as its N source, which has been associated with increased seed oil content and improved drought tolerance, while specific lipid percentages may vary depending on environmental conditions and cultivation practices (Li-Beisson et al., 2013). Duckweed responds optimally to NH_4_^+^ over NO_3_^-^, with up to a 35% increase in TAG content and strong adaptation to NH_4_^+^ -rich wastewater environments (Tian et al., 2021; Liang et al., 2022). These findings highlight the importance of optimizing N fertilization strategies to balance lipid yield, growth, and N agronomic efficiency.

### 3.4 Interactions with other nutrients

N partitioning in high-lipid crops is often intertwined with interactions with other nutrients, such as sulfur (S) and phosphorus (P). Sulphur regulates N metabolism and lipid biosynthesis, with S deficiency leading to reduced protein synthesis and altered lipid composition (Anjum et al., 2012). Similarly, synergistic effects of N and P enhance lipid production and crop resilience, as seen in microalgae and oilseed crops (Huang et al., 2019). For example, in rapeseed, combined N and P application increased lipid content by 20% compared to N application alone, highlighting the importance of integrated nutrient management for optimizing lipid biosynthesis (Peng et al., 2023).

## 4 Redox regulation in the photosynthetic tissues and its influence in non-seed lipid storage

The shift from conventional lipid storage in seeds to vegetative tissues requires a deeper understanding of the interplay between redox potential and metabolic regulation. Redox regulation is central to managing cellular energy and oxidative stress, particularly in photosynthetic tissues where light-driven electron transport generates ROS. This section explores the mechanisms of redox regulation, its impact on lipid biosynthesis, and its role in plant stress responses, with a focus on non-seed lipid storage.

### 4.1 Redox homeostasis in photosynthesis

During photosynthesis, ROS are produced at multiple sites (**Figure 1**). PSII generates singlet oxygen through the excitation of chlorophyll. PSI produces superoxide radicals (O_2_^-^·) *via* the Mehler reaction. Photorespiration generates hydrogen peroxide (H_2_O_2_) in peroxisomes through glycolate oxidase activity.

These ROS molecules act as signaling intermediates, modulating metabolic pathways and stress responses. Under optimal conditions, ROS production is balanced by antioxidant systems, maintaining redox homeostasis. However, excessive ROS accumulation can damage lipids, proteins, and DNA, impairing cellular function (Apel and Hirt, 2004).

### 4.2 Redox regulation of lipid biosynthesis

The chloroplast ETC supplies much of the reducing power, with ferredoxin (Fdx) playing a key role in transferring electrons to NADP^+^, forming NADPH (Shikanai, 2007). This NADPH is utilized by ACCase and the FAS complex for FA biosynthesis (Li-Beisson et al., 2013).

Redox regulation also influences FA desaturation, a critical step in lipid biosynthesis. FA desaturases require Fdx as an electron donor, linking redox status to membrane fluidity and stress tolerance (Shanklin and Cahoon, 1998).

### 4.3 ROS scavenging and lipid protection

To mitigate ROS-induced damage, plants employ a suite of antioxidant systems, including the superoxide dismutase-ascorbate-glutathione (SOD-AsA-GSH) cycle (Foyer-Halliwell-Asada pathway). This cycle involves (**Figure 2**): SOD converts O_2_^-^· to H_2_O_2_; ascorbate peroxidase reduces H_2_O_2_ to water using AsA as an electron donor; monodehydroascorbate reductase and dehydroascorbate reductase regenerate AsA from its oxidized forms; and glutathione reductase maintains GSH levels, essential for ROS scavenging and redox signaling (Polle, 2001).

In high-lipid plants, ROS scavenging is particularly important due to the increased metabolic activity associated with lipid biosynthesis. For example, in high-lipid ryegrass, elevated SOD activity and AsA levels, alongside reduced expression of cytosolic L-AsA oxidase and APX, reflect the need to regulate ROS and conserve reducing equivalents for FA biosynthesis (Winichayakul et al., 2022). These adaptations help maintain redox balance while supporting lipid accumulation.

### 4.4 Redox regulation of TAG turnover

Leaf TAG is used as a short-term storage intermediate of thylakoid lipid during ongoing membrane turnover, remodeling, and senescence (Slocombe et al., 2009; James et al., 2010). TAG turnover in leaves is dynamic, particularly during vegetative growth and senescence, with rates of ~1.2 mol% per minute in Arabidopsis under 22°C conditions (Bao et al., 2000; Troncoso-Ponce et al., 2013). TAG recycling during high energy demand can increase β-oxidation activity in peroxisomes, potentially elevating ROS production (**Figure 1**; Yu et al., 2019). In Arabidopsis, SDP1 lipase-mediated TAG degradation supplies C for dark survival (Fan et al., 2017), while lipid peroxidation products (e.g., jasmonates) regulate senescence timing (Yu et al., 2020a). Protecting the TAG β-oxidation may improve vegetative oil yield and reduce ROS-induced damage. Strategies include: engineering cysteine oleosin to enhance TAG-associated protein cross-linking, stabilizing lipid droplets (Winichayakul et al., 2013); RNAi suppression of SDP1 to reduce TAG lipase activity, minimizing TAG turnover (Kelly et al., 2013; Fan et al., 2014), and disrupting CGI-58 and PXA1 to inhibit peroxisomal TAG breakdown, reducing ROS production (Park et al., 2013).

## 5 Environmental impacts and field evaluation

High-lipid crops offer transformative potential for sustainable agriculture and bioenergy production. However, their successful deployment depends on adaptability to diverse environmental conditions, efficient agronomic management, and minimal ecological trade-offs. This section reviews the performance of high-lipid crops under key abiotic stresses—temperature, light, and water availability—and discusses their ecological and agronomic implications.

### 5.1 Temperature effects on lipid synthesis

Temperature fluctuations significantly influence lipid synthesis and plant performance (**Table 2**). Cooler temperatures promote polyunsaturated FA synthesis, such as increased 18:3 at the expense of 18:2, maintaining plasma membrane fluidity (Zheng et al., 2011). However, chilling temperatures can impair photosynthesis and induce oxidative damage, particularly in warm-climate species (Allen and Ort, 2001).

**Table 2:** Lipid metabolism in response to abiotic stresses in plants.

Stress type	Effect on C/N partitioning	Impact on lipid biosynthesis	Key lipid classes involved	Enzymes/pathways affected	Impact on plant response	Citations
Heat	Increased respiration raises C consumption. N may shift to protein synthesis and stress-related metabolites.	C is allocated to protective lipids, leading to TAG accumulation.	Saturated galactolipids, phospholipids (PC, PE), TAG.	FA catabolism aids efficient stomatal opening via Ca²^+^-dependent calmodulin-binding kinase.	Lipid composition changes maintain membrane fluidity, TAGs accumulate as energy reserves, and heat shock proteins are upregulated.	Oksala et al., 2014; Mueller et al., 2017; Yurchenko et al., 2018; Lu et al., 2020; Shiva et al., 2020; Rahman et al., 2022.
Cold	Reduced metabolic rate leads to C accumulation as sugars and lipids. N is redirected to stress-related amino acids.	Unsaturated FA accumulate to maintain membrane rigidity but also trigger lipid peroxidation.	Unsaturated FA, and PC, DGDG.	Cold-responsive genes are activated, leading to an increase in desaturases like FAD2.	Unsaturated FAs enhance membrane stability. 18:3, a precursor for JA synthesis, regulates cold tolerance gene expression via MeJA.	Allen and Ort, 2001; Zheng et al., 2011; Laura et al., 2018; Wang et al., 2020; Jurrakko et al., 2021.
High light	Increased C flux out of chloroplasts. Oxidative stress disrupts N metabolism, shifting C toward stress mitigation.	Guard cell TAG provides ATP for stomatal opening and supports ER lipid synthesis.	Phospholipids (PC, PE), TAG, and antioxidants.	TAG recycling activates lipid peroxidation, lipoxygenases, and FA desaturation, altering phytohormone dynamics.	Lipid biosynthesis supports membrane protection, energy storage, and cellular integrity under excess light, while antioxidant production increases.	Fan et al., 2017; Olzmann and Carvalho, 2019; Lu et al., 2020; Yu et al., 2020a; Obaseki et al., 2024.
Low light	Reduced CO_2_ fixation causes C limitation. N is reallocated to essential metabolic function.	Limited C reduces lipid biosynthesis, but essential phospholipids remain. Lower stress increases FA in leaves.	Phospholipids (PC, PE), galactolipids, Free FA.	Enzymes in FA synthesis and C allocation, such as plastidic ACCase, WRI1, LEC2, and sugar signaling genes, are downregulated.	Lipid production for membrane growth is reduced, with altered composition to conserve energy.	Miller et al., 2017; Yu et al., 2020a; Kuczynski et al., 2022; Winichayakul et al., 2022; Han et al., 2025.
Drought	C is redirected from sugars to lipid storage. Amino acid and protein biosynthesis decline due to limited N.	TAGs increase as energy reserves, while FA composition shifts to minimize water loss.	TAG, galactolipids, phospholipids, and sphingolipids increase, with a rise in unsaturated FAs.	DGAT and desaturases are upregulated for lipid production, while ACCase and FAS are downregulated, and phospholipase D is upregulated. ROS production is induced, leading to lipid peroxidation.	Lipid accumulation mitigates dehydration and osmotic stress, providing energy under water stress. Membrane permeability is reduced, enhancing stress tolerance. APX and GR levels increase to protect plants.	Ratnayaka et al., 2003; Gigon et al., 2004; Wang, 2005; Zhang et al., 2014; Beechey-Gradwell et al., 2018; Sharma et al., 2023; Yin et al., 2024.
Salt	N is redirected to stress metabolites like proline, while excess C supports lipid synthesis.	N limitation increases lipid accumulation, mainly as TAGs storing excess C.	Phospholipids (PC, PE), TAG, and betaine lipids.	Enzymes in N metabolism (e.g., nitrate reductase) and lipid biosynthesis (e.g., phospholipase D) are altered, with increased levels of ABA and JA.	Stomata close, impacting plant growth. Lipid accumulation supports membrane stability and stress tolerance, while TAG energy reserves buffer against osmotic imbalance.	Apel and Hirt, 2004; Munns, 2005; Zhang et al., 2014; Yu et al., 2020b.
Oxidative Stress	C is shifted to protective metabolites (e.g. antioxidants) and N to stress-response proteins.	C and N are redirected to protect against lipid peroxidation, increasing phospholipid synthesis and altering FA composition.	Phospholipids (PC, PE), galactolipids, TAGs, and antioxidants.	Lipid oxidation pathways are activated, with enzymes like lipoxygenase and phospholipase A2 preventing oxidative damage.	Lipid rearrangements protect membranes from ROS-induced damage, while increased antioxidants mitigate oxidative stress. Oxidized proteins are degraded *via* autophagy.	Ratnayaka et al., 2003; Chaki et al., 2020; Qamer et al., 2021.

ACCase, acetyl-CoA carboxylase; APX, ascorbate peroxidase; DGAT, acyl-CoA diacylglycerol acyltransferase; DGDG, digalactosyl diacylglycerol; ER, endoplasmic reticulum; FA, fatty acid; FAD2, FA desaturase 2; GR, glutathione reductase; JA, jasmonate; LEC2, leafy cotyledon 2; MeJA, methyljasmonate; PC, phosphatidylcholine; PE, phosphatidylethanolamine; ROS, reactive oxygen species; TAG, triacylglycerol; WRI1, wrinkled 1.

Recent advances in cold acclimation research have identified strategies to enhance cold resilience. For example, plasma membrane-localized proteins reduce lipid peroxidation and ROS generation in cold-resilient crops (Juurakko et al., 2021). Antifreeze proteins from perennial ryegrass, featuring leucine-rich repeat domains and a β-helical carboxyl ice-binding domain, improve freeze protection (Lauersen et al., 2011). Symbiotic relationships with plant-associated microbiomes also promote growth and reduce lipid peroxidation under cold stress (Zhu et al., 2010; Osman et al., 2013).

In contrast, warm climates stimulate faster growth and heightened metabolic activity, enhancing the biosynthesis of specific lipids like TAGs (Mueller et al., 2017). TAG accumulation in vegetative tissues may help plants cope with abiotic stress through lipase-mediated lipid remodeling (Lu et al., 2020). However, high leaf-oil Arabidopsis lines exhibit increased susceptibility to heat stress (Yurchenko et al., 2018), highlighting the complexity of lipid metabolism under thermal stress.

Extreme heat impairs membrane stability and lipid functionality through oxidative stress and lipid peroxidation (Shiva et al., 2020). Field trials of high-lipid cool-season perennial ryegrass in Missouri, USA, revealed a decline in foliar FA accumulation under summer conditions (Beechey-Gradwell et al., 2022), potentially linked to rapid TAG turnover to support heat-induced stomatal opening (Korte et al., 2023). These findings underscore the need for multi-site validation of high-lipid technologies and exploration of their application in warm-climate forage species, which often produce heat-shock proteins and lipid antioxidants to mitigate heat-induced damage (Oksala et al., 2014; Rahman et al., 2022).

### 5.2 Light impacts on lipid biosynthesis

Light is a critical regulator of lipid biosynthesis, particularly in photosynthetic tissues where C and energy derived from photosynthesis play a central role (Fan et al., 2017; Lu et al., 2020). Seasonal variations in light intensity, day length, and spectral composition significantly influence lipid metabolism. Low light intensity, shorter day lengths, and a higher proportion of red and infrared light in winter can limit photosynthesis and growth. Conversely, high light intensity, longer day lengths, and a higher proportion of blue and ultraviolet wavelengths in summer support robust photosynthesis but may induce stress under excessive light.

Yu et al. (2020a) demonstrated how plants fine-tune FA and glycerolipid biosynthesis in response to long-term changes in light conditions (**Table 2**). Arabidopsis mutants defective in glycerolipid biosynthesis (*tdg1*) exhibited altered growth patterns and impaired thylakoid membrane remodeling under high light. Excess light stress can lead to ROS production, with phospholipids and galactolipids degraded to provide substrates for TAG synthesis as a protective mechanism (Olzmann and Carvalho, 2019; Obaseki et al., 2024). TAG accumulation in chloroplast-associated lipid droplets may prevent lipotoxicity from oxidative damage to FAs.

Low-light conditions reduce ROS-mediated FA breakdown, allowing greater accumulation of free FAs in leaf tissues. However, low light can disrupt the balance between photosynthate production and utilization. Plastidic ACCase is regulated at transcriptional and posttranslational levels, underscoring its role in plant acclimation to changing irradiance (Miller et al., 2017; Yu et al., 2020a). WRI1, a key transcriptional regulator of lipid biosynthesis, integrates environmental signals and developmental cues to synchronize FA production with plant energy status (Kuczynski et al., 2022; Han et al., 2025). Transcriptomic analyses further reveal the interconnected regulation of genes involved in C metabolism, sugar signaling, mitochondrial respiration, and redox potential, all of which influence lipid metabolism under low light conditions (Winichayakul et al., 2022).

### 5.3 Water availability impact on lipid biosynthesis

Water stress, whether from drought, salinity, or flooding, significantly alters lipid composition and biosynthesis. While moderate water stress can trigger adaptive lipid responses, severe or prolonged stress typically suppresses overall lipid production, impairing growth, seed development, and stress tolerance.

Water deficit induces ROS production, leading to lipid peroxidation (**Table 2**). This triggers signaling pathways that modulate lipid biosynthesis (Sharma et al., 2023). Water scarcity limits the availability of energy and NADPH required for FA biosynthesis in plastids, reducing overall lipid production. In Arabidopsis, total leaf lipid content decreased progressively under drought, although plants exhibited cellular adaptations, such as increased galactolipid ratios and FA unsaturation (Gigon et al., 2004). While key enzymes like ACCase and FAS are often downregulated under drought, phospholipase Dϵ activity increases, generating phosphatidic acid, which participates in osmotic stress responses and cellular signaling (Wang, 2005; Sharma et al., 2023).

Recent studies highlight the role of altered FA composition in drought acclimation. For example, Yin et al. (2024) reported that high 18:3 levels contributed to drought tolerance in maize, while 16:2 and 16:3 were more critical for drought recovery. Beechey-Gradwell et al. (2018) found that high-lipid perennial ryegrass exhibited greater regrowth and 16% higher WUE under limited water supply compared to controls. These findings suggest that high vegetative-lipid technology may enhance drought tolerance, although further field testing is needed to confirm these effects (Blum, 2005).

Salinity stress similarly disrupts water uptake and generally leads to reduced transpiration due to stomatal closure, leading to osmotic stress and enhanced photorespiration (Munns, 2005). Photorespiration generates significant ROS, which can damage lipids and other cellular components (Apel and Hirt, 2004). The balance between ROS production and scavenging enzyme activity determines whether signaling or damage occurs (Zhang et al., 2014). For instance, increased production of ascorbate peroxidase and glutathione reductase protects plants from oxidative stress under drought and salinity (**Table 2**, Ratnayaka et al., 2003; Chaki et al., 2020; Qamer et al., 2021).

### 5.4 Crop selection and field evaluation

The development and deployment of high-lipid crops require a dual focus on crop selection and field evaluation. Crop selection depends on the intended application, with ongoing debate about the impact of using food crops as biofuel feedstocks (Singh et al., 2023). Agricultural residues and non-food feedstocks like algae and duckweed offer competitive alternatives (Ullmann and Grimm, 2021; Yang, 2022). Algae and duckweeds exhibit high lipid content under controlled conditions, but their performance in open pond systems or natural aquatic environments requires validation to ensure consistent yields (Wang et al., 2024a). **Table 3** summarizes key findings from laboratory to agricultural and bioenergy applications, including performance metrics for scaling cultivation.

**Table 3:** Field performance and evaluation of bioenergy crops, algae, and duckweeds.

Crop species	Location	Key findings	Performance metrics	Citations
Algae	Arizona USA	Feasibility of large-scale lipid production in open pond systems.	Lipid yields of 20-30% DW; affected by light, temperature, and nutrients.	Ullmann and Grimm, 2021; Wang et al., 2024a.
Camelina	Hertfordshire, UK; Manitoba, Canada; University of Nebraska, USA	Stable traits under low-input conditions.	Overexpressing EPA/DHA pathways boosts *n*-3 LC-PUFAs, significantly altering seed fatty acid composition and mildly affecting seed TAG profile.	Usher et al., 2015 & 2017; Han et al., 2020 & 2022.
Duckweeds	Global aquatic system	Rapid growth, high lipid content; thrives in nutrient-rich wastewater. GE advances boost TAG production and growth rates.	Lipid content: 20-35% DW (optimal conditions); biomass doubles in 1–2 days. ~8.7% DW TAG in GE *Lemna japonica*; resilient to high salinity and heavy metals.	Liang et al., 2022; Yang, 2022.
Energycane	Florida, Louisiana USA	High biomass and sugar content; lipid engineering potential in stems. Adapts well to drier, cooler climates.	Biomass yields: 30–50 Mg/ha; stem lipid content: 1-3% DW. GE energycane achieved TAG ~3.85% leaf DW, TFA ~8.4% DW, and stem TAG ~1.14% DW.	Luo et al., 2022; Cao et al., 2023.
Maize (corn)	Midwest USA	High-lipid varieties for bioenergy; dual-use potential (food and fuel).	Grain lipid: 4-6% DW; biomass yields: 10–15 Mg/ha. A transgenic event increased leaf oil by 79%. High-oil maize breeding showed consistent low grain yields.	Moose and Below, 2009; Alameldin et al., 2017; Li et al., 2023.
Miscanthus	Illinois USA, Europe	High biomass yields, low input needs; lipid engineering potential at the expense of polysaccharides and lignin.	Biomass yields: 20–40 Mg/ha; current lipid content: 1-3% DW in stems and leaves.	Wang et al., 2008; Heaton et al., 2010.
Perennial ryegrass (Lolium)	New Zealand, Missouri USA	As a mini-sward, 16% higher WUE and improve regrowth under drought.	Biomass yields: 1–4 Mg/ha; leaf TFA: 5-5.8% DW, with GE increase of 1-1.2 kJ/g DW. Engineered ryegrass showed reduced FA accumulation in summer heat.	Beechey-Gradwell et al., 2018 & 2022; Alckmin et al., 2022.
Sorghum	Texas, Kansas, Eastern Nebraska, California USA	High biomass, drought-tolerant; potential for leaf lipid accumulation.	Biomass yields: 15–25 Mg/ha; TFA: 3-5% DW in vegetative tissues. GE sorghum achieved 6.9% DW TFA, 4.6% DW TAG, with TAG in top events at 5.5% DW in leaves and 3.5% DW in stems. GE sorghum produced 1.2% DW 4-HBA but saw a 15% biomass reduction.	Mullet et al., 2014; Vanhercke et al., 2018; Lin et al., 2022; Park et al., 2025.
Sugarcane	Brazil, India, USA	High biomass yields; potential for lipid accumulation in stems and leaves.	Biomass yields: 60–100 Mg/ha; lipid content: 1-3% DW in vegetative tissues. GE sugarcane achieved 8% DW TAG and 13% DW TFA in leaves, and 4.3% DW TAG in stems.	Matsuoka et al., 2014; Zale et al., 2016; Parajuli et al., 2020.
Switchgrass (Panicum)	Great Plains, California USA	High biomass, drought-tolerant; potential for leaf lipid accumulation.	Biomass yields: 10–20 Mg/ha; lipid content: 2-4% DW in vegetative tissues. GE switchgrass reduced recalcitrance, increasing biomass and saccharification.	Sage et al., 2011 & 2015; Eudes et al., 2023.
Tobacco	USA, Europe, Australia	High lipid content in leaves and stems; adaptable to diverse climates and stress conditions.	Lipid content: 15-20% DW in engineered varieties; rapid growth with high biomass yields.	Vanhercke et al., 2019; Zhou et al., 2020; Chu et al., 2022.

EPA, eicosopentanoic acid; DHA, docosohexanoic acid; GE, genetic engineering; 4-HBA, 4-hydroxybenzoic acid; LC-PUFA, long-chain polyunsaturate fatty acid; TFA, total fatty acid.

High-lipid crops must demonstrate adaptability to a wide range of environmental conditions, including variations in temperature, light, water availability, and soil quality. For example, perennial ryegrass has shown promise in cool climates but exhibits reduced lipid accumulation under summer heat, highlighting the need for climate-specific optimization (Beechey-Gradwell et al., 2022). Camelina have demonstrated resilience to drought and marginal soils, making them suitable for cultivation in water-limited regions (Usher et al., 2015 & 2017; Han et al., 2020). Tobacco, traditionally grown for nicotine production, has emerged as a promising high-lipid crop due to its rapid growth and adaptability to diverse climates including drought and high salinity, making it a robust option for cultivation in challenging environments (Zhou et al., 2020). Field trials have shown that tobacco can accumulate significant lipid content, reaching up to 15-20% DW in its leaves and stems, particularly under stress conditions, making it a viable candidate for bioenergy production (Vanhercke et al., 2019). However, at these levels of vegetative lipid accumulation, tobacco typically experiences severe growth penalties, limiting its potential for commercialization.

Several genetic and biotechnological strategies have been explored to enhance vegetative oil production in crops such as maize, sorghum, and sugarcane, due to their adaptability to diverse environmental conditions, including drought and high temperatures (**Table 3**) (Dida, 2024). Maize engineered for increased lipid content in seeds and vegetative tissues often shows reduced grain yield (Moose and Below, 2009; Alameldin et al., 2017; Li et al., 2023). The ‘push, pull, protect’ (3P) GM strategy (Xu and Shanklin, 2016) achieved TAG concentrations of up to 8.4% DW in sorghum leaves, though trait stability and growth effects in subsequent generations were not assessed due to multiple T-DNA insertions (Vanhercke et al., 2018). Recently, Park et al. (2025) reported successful field trials of sorghum engineered for high vegetative oil content, achieving 5.5% DW in leaves and 3.5% DW in stems without growth penalties. This approach combined the 3P strategy with medium-chain FA generation, contrasting with growth inhibition observed in engineered sugarcane and energycane (Matsuoka et al., 2014; Zale et al., 2016; Parajuli et al., 2020; Luo et al., 2022; Cao et al., 2023). Notably, engineered sorghum lines exhibited higher DGAT1 expression than WRI1, suggesting sufficient DGAT1 activity to enhance FA biosynthesis flux through the ER glycerolipid pathway. Imbalances between WRI1 induction and DGAT activity can lead to toxic free FA accumulation, a key bottleneck in high-lipid crop development (Yang et al., 2015; Parajuli et al., 2020; Kannan et al., 2022).

C4 perennial species such as Miscanthus spp., switchgrass, and prairie cordgrass are also gaining recognition as bioenergy feedstocks in cool climates. These crops benefit from the abundance of arable land in the Northern Hemisphere and the growing demand for biofuels (Sage et al., 2011 & 2015). These grasses hold potential for lipid engineering at the expense of polysaccharides (Eudes et al., 2023). Breeding efforts must prioritize cold-tolerant traits to ensure robust growth in cooler climates (Sage et al., 2015). For instance, pyruvate phosphate dikinase, a key enzyme in C4 photosynthesis, exhibits higher protein content and activity in Miscanthus x giganteus under cooler conditions, enhancing photosynthetic capacity (Wang et al., 2008; Heaton et al., 2010). Transgenic maize overexpressing this enzyme showed improved photosynthetic rates at low temperatures, highlighting its potential for expanding bioenergy crop suitability (Thomashow et al., 2001).

## 6 Summary and future directions

Integrating TAG storage into non-seed tissues holds great promise for enhancing crop energy density. The degree of vegetative TAG accumulation varies across species, influenced by the biotechnological approach employed. While some plants experience growth penalties, others exhibit enhanced growth at different lipid accumulation levels. Although improved photosynthetic efficiency in high-lipid plants is thought to result from mitigating feedback inhibition through the dynamic redirection of carbohydrate flux, this metabolic shift is far more complex, involving changes in carbon and nitrogen partitioning, as well as redox homeostasis, affecting plant responses to diverse environmental conditions.

To fully realize the agricultural potential of lipid biosynthesis in crop biomass, several key research directions should be prioritized:

1. *Integrative multi-omics approaches*
    This integrative approach could identify novel targets for enhancing crop resilience and productivity in diverse field conditions.
2. *Environmental stress adaptation*
    Investigating how lipid accumulation influences stress signaling and energy metabolism will be crucial for developing cultivars with improved tolerance to environmental stresses.
3. *Carbon-nitrogen interactions*
    Understanding the interplay between C and N metabolism, particularly how N availability affects lipid biosynthesis and redox balance, will inform breeding strategies aimed at optimizing NUE while maintaining high lipid yields.
4. *Field trials and real-world applications*
    Conducting extensive field trials under varying environmental conditions is essential for validating the benefits observed in controlled settings. These trials will refine cultivation practices and assess the feasibility of scaling up lipid biosynthesis technologies for large-scale agricultural deployment.
5. *Alternative crop* sp*ecies and systems*
    Expanding research to include other crop species, such as C4 plants, duckweeds, and microalgae, could broaden the applications of these technologies. Duckweed and microalgae, for examples, have demonstrated promising lipid accumulation under stress conditions, making them potential candidates for biofuel production and carbon sequestration.

By addressing these key areas, future research can bridge the gap between laboratory breakthroughs and field implementation, paving the way for sustainable agricultural innovations that meet global food and energy demands.
